# Magnetic field inhomogeneities due to CO_2_ incubator shelves: a source of experimental confounding and variability?

**DOI:** 10.1098/rsos.172095

**Published:** 2018-02-14

**Authors:** L. Makinistian, I. Belyaev

**Affiliations:** 1 Department of Radiobiology, Cancer Research Institute, Biomedical Center, Slovak Academy of Sciences, Bratislava, Slovakia; 2 Department of Physics and Instituto de Física Aplicada (INFAP), Universidad Nacional de San Luis, Consejo Nacional de Investigaciones Científicas y Técnicas, Ejército de los Andes 950, 5700 San Luis, Argentina; 3 Laboratory of Radiobiology, General Physics Institute, Russian Academy of Sciences, Moscow, Russia

**Keywords:** background fields of incubators, biological effects of weak static magnetic fields, strain-induced magnetization of austenitic stainless steel

## Abstract

A thorough assessment of the static magnetic field (SMF) inside a CO_2_ incubator allowed us to identify non-negligible inhomogeneities close to the floor, ceiling, walls and the door. Given that incubator's shelves are made of a non-magnetic stainless steel alloy, we did not expect any important effect of them on the SMF. Surprisingly, we did find relatively strong distortion of the SMF due to shelves. Indeed, our high-resolution maps of the SMF revealed that distortion is such that field intensities differing by a factor of up to 36 were measured on the surface of the shelf at locations only few millimetres apart from each other. Furthermore, the most intense of these fields was around five times greater than the ones found inside the incubator (without the metallic shelves in), while the lowest one was around 10 times lower, reaching the so-called hypomagnetic field range. Our findings, together with a survey of the literature on biological effects of hypomagnetic fields, soundly support the idea that SMF inhomogeneities inside incubators, especially due to shelves' holes, are a potential source of confounding and variability in experiments with cell cultures kept in an incubator.

## 1. Introduction

According to McDonald [1], a confounding variable is a variable other than the independent variable that we are interested in, that may affect the dependent variable, i.e. the endpoint of our experiment. Hence, a key aspect of experimental design is the identification of possible confounding variables and some kind of control of them. The main message of this work is that there might be a confounding variable (almost) completely unaccounted for in biology laboratories working with incubators: the static magnetic fields (SMFs), and their inhomogeneities throughout the volume of incubators, especially on the surface of the shelves, where cell cultures are commonly kept in biological experiments.

The matter has, in fact, received some attention during the last years among magnetobiology researchers, who have discussed and measured direct current (DC, or ‘static’) and alternated current (AC) background magnetic fields (MFs) inside CO_2_ incubators. Hansson Mild *et al*. [2] measured AC fields inside an incubator and discussed the importance of the ones generated by the incubator's fan, also commenting on equipment placed near the incubator being a possible source of stray fields. In line with that discussion, Gresits *et al*. [3] measured AC fields not only in a CO_2_ incubator, but also near a thermostatic water bath and a laboratory shaker table, finding non-negligible intensities, with significant variations over relatively short distances. To the best of our knowledge, the most extensive work on CO_2_ incubators was done by Portelli *et al*. [4], who measured both DC and AC fields inside of 21 different incubators. These fields are of particular importance in experiments of weak DC and/or extremely low frequency MFs, where the field intensities under study are of the same order of magnitude as the fields typically present inside incubators, clearly posing them as a potential confounding variable, and also making replication by independent laboratories more difficult [5].

As stated above, we suggest that background fields should be of concern to all researchers working with incubators, not only the ones devoted to magnetobiology. In the following sections, we make our case by: (i) presenting a thorough assessment of the SMFs inside a typical CO_2_ incubator including, for the first time, a high-resolution mapping of the fields near several holes of a standard, stainless steel (SS) shelf and (ii) discussing our measurements in the context of an up-to-date survey of the literature on biological effects of weak SMFs, including the so-called hypomagnetic fields.

## 2. Material and methods

**Figure 1.**
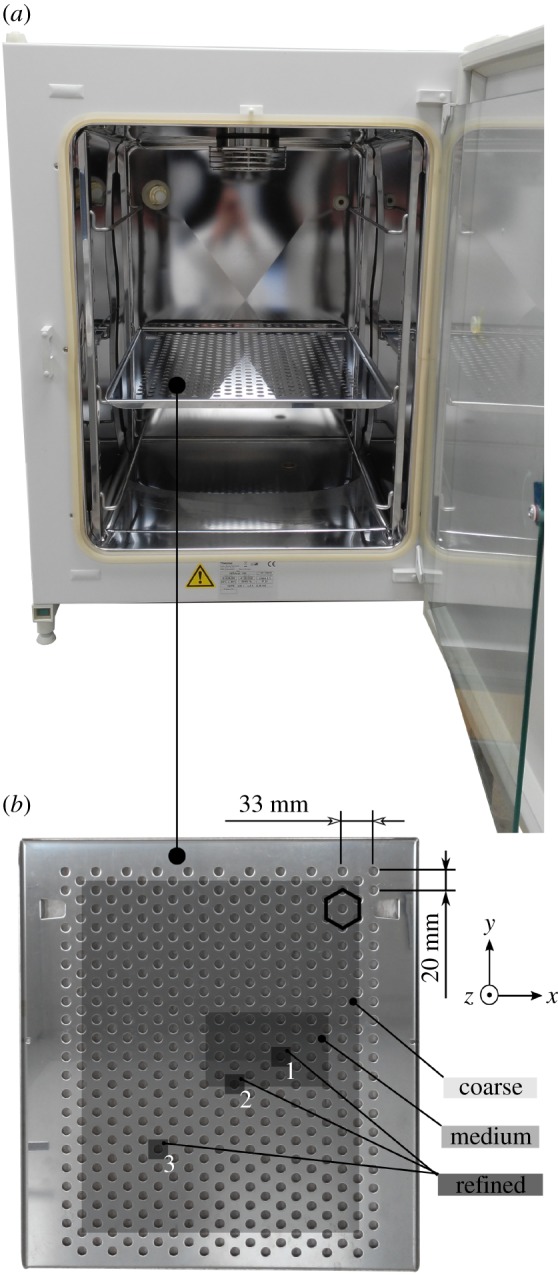
(*a*) The HERACell 150i CO_2_ Incubator (Thermo Fisher Scientific, MA) studied in this work and (*b*) one of its shelves. The grey-shaded areas indicate the locations of the coarse, medium and refined measuring grids. The small hexagon in the upper right corner is a visual aid for recognizing the holes pattern on the shelf.

**Figure 2.**
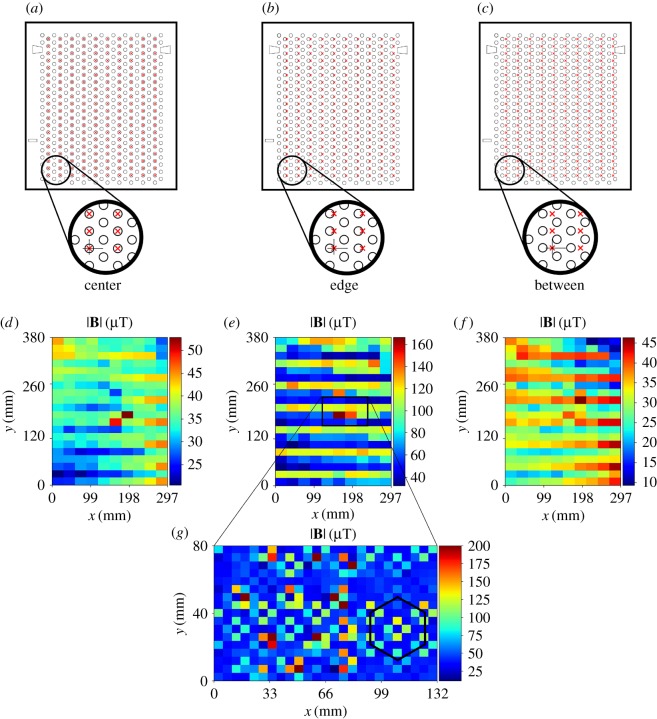
(*a*–*c*) Schematics of a metallic shelf with the coarse measuring grid overlapped to it. Red crosses indicate the position of measurements. (*d*–*f*) MF strength for the grid aligned to the centre, edge and between the holes, respectively. The area delimited by the black rectangle in (*e*) was further investigated, (*g*) with a medium, 5.5 × 5 mm grid. The black hexagon has the exact same dimensions as the one in the upper right corner of figure 1*b*. Although there are a few pixels in (*g*) in the range of 200–415 µT (dark-brown pixels), colour scale maximum was limited to 200 µT to enhance contrast of the image, thus making the hexagonal pattern visible.

All measurements were performed in a HERACell 150i CO_2_ Incubator (Thermo Fisher Scientific, MA; figure 1*a*). Metallic shelves provided with the incubator by the manufacturer were made of the austenitic SS AISI 304, an extremely common and well-known alloy. We also used an 8 mm thick 420 × 465 mm plastic shelf made of polymethyl methacrylate (PMMA). Measurements were performed with an HMC5883 L 3-axis magnetometer (Honeywell, New Jersey, NY) connected to a personal computer, as detailed elsewhere [6]. For this work, each independent axis of the sensor was calibrated against a TM75-41 magnetometer (Izmiran, Fryazino, Russia). Each of the MF maps presented in the next section was measured by manually locating the sensor in each of the points of a regular grid. Upon the assumption that the holes of the metallic shelves could be a source of distortion of the SMFs inside the incubator, we decided to use a grid with a periodicity that would match that of the shelf's holes, so that all the measurements of a given map would be performed in equivalent points of the holes' lattice. Hence, according to the dimensions indicated in figure 1*b*, we defined one first (coarse) grid with a unit of 33 × 20 mm^2^, which covered almost all the usable area of the shelf. Also, suspecting that the field at the centre of the holes could be different from that at the edges, or from that between holes, we measured three maps with the coarse grid, shifting its position just 5 mm to the right each time (holes' diameter was 10 mm; figure 2*a*–*c*). Measurements with the coarse grid led us to a medium grid which, in term, led us to further refinement. Locations of the grids on the shelf are shown as grey rectangular areas in figure 1*b*, and further details are presented in table 1, which shows that the smallest of our pixels were 1 mm^2^. While the chip that contains the three orthogonal sensors of our magnetometer has external dimensions of 3.0 × 3.0 × 0.9 mm, we considered it reasonable to assume that the sensors themselves are confined in an area smaller than 1 mm^2^, given the presence of the plastic packaging encapsulating the electronics, plus the fact that circuitry auxiliary to the sensors is also contained inside the same chip (a multiplexer, an analogue to a digital converter and a control unit, among others).

**Table 1.** Grids used in this work.

	coarse	medium	refined
area (mm^2^)	297 × 380	132 × 80	22 × 22
pixel size (mm^2^)	33 × 20	5.5 × 5	1 × 1
number of pixels	10 × 20 (200)	25 × 17 (425)	23 × 23 (529)
approximate uncertainty in location of sensor (mm)	±2	±1	±0.5

## 3. Results

**Figure 3.**
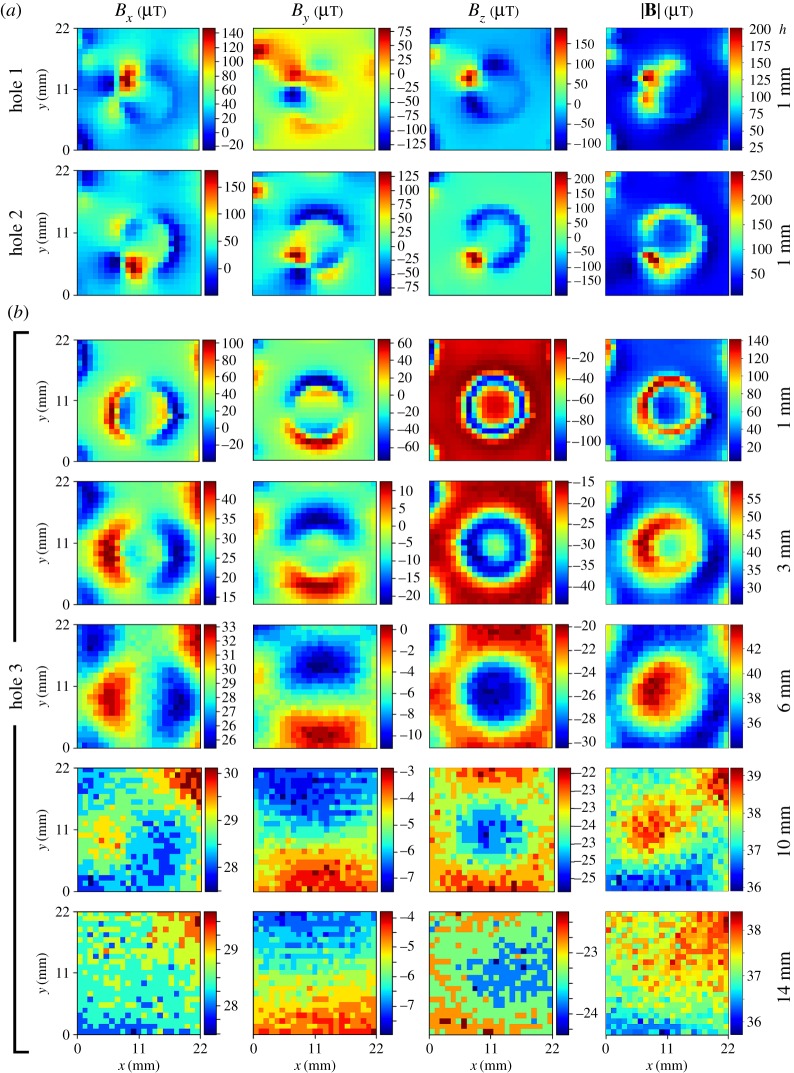
(*a*) MF components (*B_x_*, *B_y_* and B_z_) and total strength (|**B**|) at 1 mm over holes 1 and 2. (*b*) The same as (*a*) for hole 3, but for several distances, *h*, from the surface (*h* = 1, 3, 6, 10 and 14 mm).

Figure 2*d* shows the total MF strength, |**B**|, measured on the metallic shelf located at the middle of the incubator (sixth level out of 11 possible) and with the grid's points coinciding with the centre of holes (figure 2*a*). Fields were in the range of 20.5–52.9 µT. Figure 2*e* corresponds to aligning the grid to the holes' right-side edge (figure 2*b*); fields fell in the range of 32.4–166.4 µT. Lastly, measurements taken between holes (figure 2*c*,*f*), were within 6.1–49.3 µT. We observed that: (i) none of the maps showed smooth variations, meaning that there were many clearly abrupt changes in the field intensity from one pixel to the surrounding ones; (ii) the three ranges of intensities are clearly different, and they all include the value of the geomagnetic field at Bratislava, Slovak Republic (where the measurements were conducted), of approximately 48.7 µT [7]; and (iii) The highest intensities were measured with the grid aligned with the holes' edges. In summary, these observations suggested that the strongest fields are at or near the edges, but also that they do not occur at all edges (or at least not evenly along their whole perimeter), because ‘low-field’ horizontal blue bands are present in figure 2*e*. Also, these maps strongly suggested that the used grid was not fine enough (i.e. pixels were too big) to assess the fields in full detail. Therefore, we used a medium grid with pixels of 5.5 × 5 mm^2^ to explore an area (black rectangle) around the ‘hot-spots’ in figure 2*e*, and the result is shown in figure 2*g*. Even though this map's pixels had an area 24 times smaller than the one in the coarse grid, it still looked ‘pixelated’. However, it showed a noisy, but discernable, hexagonal pattern: the black hexagon, added to the map as a visual aid, has exactly the same dimensions as the one at the upper right corner of figure 1*b*, suggesting that the magnetic pattern was the same as the holes' pattern on the shelf. Upon these findings, we further refined our grid to 1 × 1 mm^2^ and explored with it three 22 × 22 mm^2^ areas centred in three different holes. At first, we located our sensor as close as possible to the surface of the shelf, at an estimated height, *h*, of 1 mm. Figure 3*a* shows the three components, *B_x_*, *B_y_*, and *B_z_*, and the total strength, |**B**|, for holes 1 and 2 (figure 1*b*). In these two maps, the MF is clearly shaped by the presence of the hole, but in an irregular way. By contrast, in the first row of figure 3*b*, the pattern of hole 3 clearly resembles the circular shape of the hole. In the succeeding rows of figure 3*b*, the same hole (number 3) was reassessed, but at greater heights (*h* = 3, 6, 10 and 14 mm). It is evident how field is homogenized as distance from the shelf increases, fading into the background field. Table 2 displays minima, maxima and differences (Δ) corresponding to all maps in figure 3.

**Table 2.** Minimum, maximum and differences (Δ) for three shelf's holes (figure 1*b*). All values are in µT, within a precision of ±2 µT.

		*B_x_*			*B_y_*			*B_z_*			|**B**|		
hole	*h* (mm)	minimum	maximum	Δ	minimum	maximum	Δ	minimum	maximum	Δ	minimum	maximum	Δ
1	1	−25.0	146.9	171.9	−134.0	80.0	214.0	−116.3	187.9	304.2	21.1	201.2	180.1
2	1	−49.5	179.6	229.1	−87.5	132.5	220.0	−194.6	220.7	415.3	7.1	257.7	250.6
3	1	−39.0	103.6	142.6	−74.5	64.6	139.1	−117.3	−2.1	115.2	4.2	141.3	137.1
3	3	13.1	44.5	31.4	−22.4	12.5	34.9	−44.0	−14.7	29.3	25.3	59.9	34.6
3	6	24.4	33.2	8.8	−11.2	0.4	11.6	−30.4	−19.8	10.6	34.0	43.9	9.9
3	10	27.5	30.1	2.6	−7.5	−2.8	4.7	−25.9	−22.3	3.6	35.9	39.2	3.3
3	14	27.5	29.7	2.2	−8.0	−3.8	4.2	−24.3	−22.3	2.0	35.7	38.4	2.7
overall		−49.5	179.6	229.1	−134.0	132.5	266.5	−194.6	220.7	415.3	4.2	257.7	253.5

**Figure 4.**
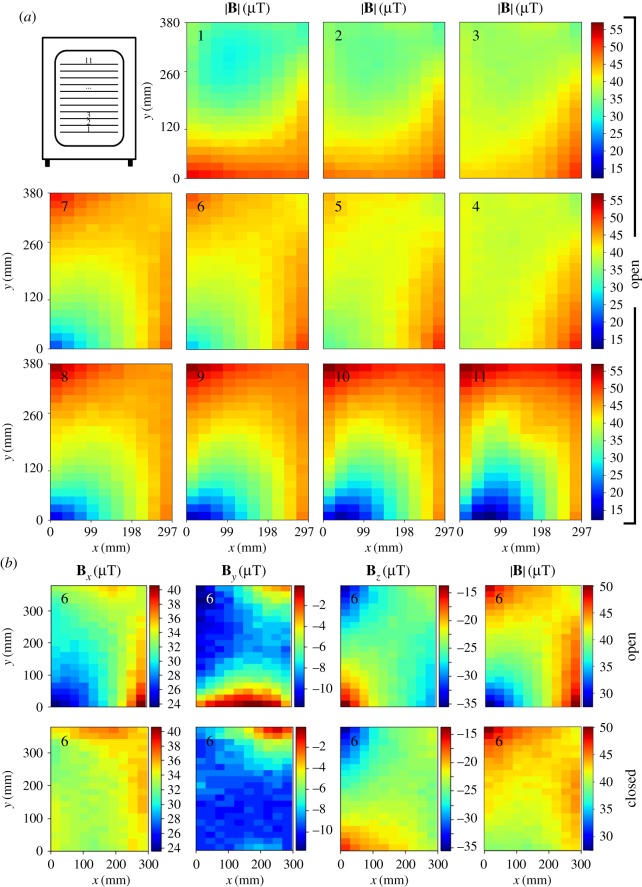
(*a*) MF total strength (|**B**|) at the 11 shelf levels of the incubator, measured on a plastic shelf and the incubator's door open. (*b*) Comparison of field's components and total strength at the sixth level, for the door open and closed.

Having determined that the metallic shelves were indeed a source of non-negligible distortion, in order to study the eventual distortion of the fields only due to the incubator's walls, ceiling and floor, all metallic shelves were removed from the incubator, the plastic shelf was positioned at each of its 11 levels and fields were assessed using the coarse grid. Figure 4*a* shows the 11 MF maps, all plotted in the same colour map scale for ease of comparison (see table 3 for numerical values). First observation to be made is that maps are smooth (i.e. only slightly ‘pixelated’): without the distortion from the metallic shelves, the coarse grid turns out to be appropriate for studying the fields inside the incubator. It is clear that variations within several centimetres are relatively subtle both in each level, and between consecutive levels (which are 4 cm apart). However, differences greater than 40 µT are observed in the upper shelves. Also, even though differences between consecutive shelves are small, the first and the eleventh levels are clearly different. Lastly, we evaluated the effect of performing the measurements either with the incubator door open, or closed (figure 4*b*). The maps clearly show that components *B_x_* and *B_y_* (and most notably this latter, perpendicular to the plane of the door) are the most affected by the closing of the door, while *B_z_* is almost unaffected. Comparing differences for the open and closed condition (see Δ for levels 6 and 6* in table 3), it is evident that closing the door has a slight homogenizing effect on the fields inside the incubator.

**Table 3.** Minimum, maximum and differences (Δ) for the 11 shelf levels of the incubator, assessed with a plastic shelf and the incubator's door open. Level 6 was also measured with the door closed (6*). All values are in µT, within a precision of ±2 µT.

shelf	*B_x_*			*B_y_*			*B_z_*			|**B**|		
level	minimum	maximum	Δ	minimum	maximum	Δ	minimum	maximum	Δ	minimum	maximum	Δ
1	10.0	38.0	28.0	−15.9	1.8	17.7	−39.0	−9.7	29.3	28.8	52.6	23.8
2	15.7	41.0	25.3	−13.1	0.9	14.0	−33.9	−14.2	19.7	32.6	49.9	17.3
3	20.1	42.4	22.3	−12.1	0.4	12.5	−30.4	−17.3	13.1	34.1	50.6	16.5
4	24.9	42.8	17.9	−10.8	0.9	11.7	−30.9	−19.8	11.1	36.3	51.2	14.9
5	27.5	41.9	14.4	−9.8	−0.1	9.7	−32.4	−18.8	13.6	33.3	50.7	17.4
6	23.6	40.6	17.0	−11.7	−0.1	11.6	−34.4	−13.7	20.7	27.4	49.9	22.5
6*	31.9	37.1	5.2	−10.3	−1.0	9.3	−35.0	−16.8	18.2	38.9	50.1	11.2
7	18.7	38.9	20.2	−14.0	−0.5	13.5	−35.0	−10.2	24.8	22.1	52.0	29.9
8	12.6	39.7	27.1	−17.2	−0.5	16.7	−35.0	−7.2	27.8	17.3	55.5	38.2
9	6.5	40.6	34.1	−20.5	0.4	20.9	−32.9	−4.6	28.3	15.3	56.2	40.9
10	−0.1	41.9	42.0	−24.2	−1.9	22.3	−36.0	−2.6	33.4	14.0	57.0	43.0
11	−5.3	41.0	46.3	−28.4	−2.4	26.0	−40.5	−2.1	38.4	12.3	56.4	44.1
overall	−5.3	42.8	48.1	−28.4	1.8	30.2	−40.5	−2.1	38.4	12.3	57.0	44.7

## 4. Discussion and conclusion

Given that incubator's shelves are made of a non-magnetic SS alloy (AISI 304, relative magnetic permeability *µ*_r _= 1), we did not expect any important effect of them on the SMF. Surprisingly, we did find relatively strong distortion of the SMF due to shelves. Thus, a first question to address is how CO_2_ incubator shelves can display the magnetizations we measured. The answer has long been known in the metallurgy industry. Manufacturing processes like folding, stamping, drilling, extruding or punching can induce an structural transition from austenite to martensite, leading to a weak magnetization of the otherwise non-magnetic SSs [8–10]. Furthermore, this effect has been studied in detail in the particular alloy of our shelves [11–13]. It is worth noting that even though the explanation of our findings is based on facts well known by the metallurgy industry, they are probably ignored by most part of the scientific community working with cell cultures, including magnetobiology researchers.

Secondly, it is fair to question the relevance of our findings: could the MF inhomogeneities that we found actually affect the outcome of an experiment through confounding or increasing the variability of a biological endpoint? While SMFs' capability of eliciting biological effects has extensively been demonstrated (e.g. see review by the World Health Organization [14]), it is also true that most of such reports dealt with fields orders of magnitude stronger than the ones we measured (i.e. from mT to several teslas). Hence, it is appropriate to retrieve here a diversity of studies with fields no greater than 415 µT (i.e. the absolute maximum among all our measurements), reporting effects on cell-free systems [15–19], genotoxicity [20–22], *in vivo* neurophysiological effects [23–26], *in vivo* sensory receptors [27], analgesia [28,29], behaviour [30–32], muscles [33], pineal gland [34,35], development [36], modulation of hydrogen peroxide production [37] and endothelial cell proliferation [38]. Furthermore, in their extensive review, Binhi & Prato [39] gathered and analysed over 130 articles on effects of fields between 0 and 10 µT (hypomagnetic fields). These effects were observed when compared with samples ‘exposed’ to the geomagnetic field, which takes values in the range of 23–64 µT, depending on the location on the Earth [40]. Moreover, an example of special interest for the present work is the study by Martino *et al*. [41] on fibrosarcoma and colorectal cancer cells, because the authors reported changes of proliferation upon differences of approximately 35–45 µT, a range that includes the ones we measured within a single shelf, for several shelves (see Δs for |**B**| at shelves 8–11 in table 3). This indicates that, even using plastic shelves, proliferation can indeed be significantly affected by the exact location of cultures on the same shelf.

A further detail to point out is that in standard multi-well plates, typical vertical distances from inside wells' bottom to the resting plane (e.g. the shelf inside an incubator) are of 3.0 mm (Thermo Scientific, MA) or 3.53 mm (Corning, NY), while under typical experimental design in Petri dishes cells can lie 1.09 mm (MatTek, MA) over the resting plane, or as close as 0.17 mm in case of glass bottom Petri dishes (Ted Pella, CA; Cellvis, CA). In table 3, we show that at a height of 1 mm, differences as high as 250.6 µT were measured within a few millimetres distance (hole 2), while at a height of 3 mm the difference was of 34.6 µT (hole 3).

In summary, we conclude that our measurements, along with the data retrieved from the literature in the preceding paragraphs, make it sensible to suggest that SMF inhomogeneities inside incubators, and especially at typical experiment location of cells regarding metallic shelves, can be a source of confounding and variability. Consequently, the use of non-metallic shelves, along with bearing in mind the exact location of cultures inside the incubator (even on the same shelf), could enhance in-lab repeatability of results throughout all disciplines working with cell cultures in incubators, regardless of their specialty.

## Data accessibility

Our data are deposited in Dryad (http://dx.doi.org/10.5061/dryad.5k6gb) [42].
